# Associations of Prolonged QTc in Sickle Cell Disease

**DOI:** 10.1371/journal.pone.0164526

**Published:** 2016-10-13

**Authors:** Julia H. Indik, Vineet Nair, Ruslan Rafikov, Iwan S. Nyotowidjojo, Jaskanwal Bisla, Mayank Kansal, Devang S. Parikh, Melissa Robinson, Anand Desai, Megha Oberoi, Akash Gupta, Taimur Abbasi, Zain Khalpey, Amit R. Patel, Roberto M. Lang, Samuel C. Dudley, Bum-Rak Choi, Joe G. N. Garcia, Roberto F. Machado, Ankit A. Desai

**Affiliations:** 1 Department of Medicine and Arizona Health Sciences Center, University of Arizona, Tucson, AZ, United States of America; 2 Department of Medicine, University of Illinois Hospitals and Health Sciences System, Chicago, IL, United States of America; 3 Department of Medicine, University of Washington, Seattle, WA, United States of America; 4 Department of Family Medicine, Creighton University Medical Center, Omaha, NE, United States of America; 5 Department of Medicine, Oakhill Hospital, Brooksville, FL, United States of America; 6 Department of Medicine, Mercy Hospital and Health Center, Chicago, IL, United States of America; 7 Department of Surgery and Arizona Health Sciences Center, University of Arizona, Tucson, AZ, United States of America; 8 Department of Medicine, University of Chicago, Chicago, IL, United States of America; 9 Lifespan Cardiovascular Institute and Brown University, Providence, RI, United States of America; University of the West Indies Faculty of Medical Sciences Mona, JAMAICA

## Abstract

Sudden death is a leading cause of mortality in sickle cell disease, implicating ventricular tachyarrhythmias. Prolonged QTc on an electrocardiogram (ECG), commonly seen with myocardial ischemia, is a known risk for polymorphic ventricular tachycardia (VT). We hypothesized that prolonged QTc is associated with mortality in sickle cell disease. ECG were analyzed from a cohort of 224 sickle patients (University of Illinois at Chicago, UIC) along with available laboratory, and echocardiographic findings, and from another cohort of 38 patients (University of Chicago, UC) for which cardiac MRI and free heme values were also measured. In the UIC cohort, QTc was potentially related to mortality with a hazard ratio (HR) of 1.22 per 10ms, (*P* = 0.015), and a HR = 3.19 (*P* = 0.045) for a QTc>480ms. In multivariate analyses, QTc remained significantly associated with survival after adjusting for inpatient ECG status (HR 1.26 per 10ms interval, *P* = 0.010) and genotype status [HR 1.21 per 10ms interval, *P* = 0.037). QTc trended toward association with mortality after adjusting for both LDH and hydroxyurea use (HR 1.21 per 10ms interval, *P* = 0.062) but was not significant after adjusting for TRV. In univariate analyses, QTc was related to markers of hemolysis including AST (*P* = 0.031), hemoglobin (*P* = 0.014), TR velocity (*P* = 0.036), higher in inpatients (*P*<0.001) and those with an SS compared to SC genotype (*P*<0.001) in the UIC cohort as well as to free heme in the UC cohort (*P* = 0.002). These findings support a relationship of prolonged QTc with hemolysis and potentially mortality in sickle cell disease.

## Introduction

Top causes of death in sickle cell disease include acute chest syndrome, pulmonary hypertension, and cardiovascular-related conditions and sudden death[1–5], the latter defined as death that occurs rapidly and unexpectedly. While significant progress has been made on understanding the link between the other causes and death, very little is known about the etiology of sudden death. As has been previously speculated[6], patients with sickle cell disease may develop fatal ventricular arrhythmias and sudden cardiac death resulting from abnormal repolarization and structural heart disease but this link has not been made to date.

Based on a growing body of literature, patients with sickle cell disease develop a dilated cardiomyopathy with preserved ejection fraction[7, 8]. While they generally do not develop macrovascular obstructive coronary artery disease[7, 8], adult sickle cell disease patients demonstrate evidence of structural heart disease with diastolic and systolic ventricular dysfunction [9], tricuspid valve regurgitation[8, 10], myocardial microvascular ischemia identified by quantitative perfusion stress test imaging[7], and presence of myocardial fibrosis revealed by both advanced cardiac MRI imaging and histology analyses of autopsy specimens[7]. These latter risk factors support the unvalidated concept of sickle cell disease cardiomyopathy. These findings may be clinically relevant as they may be associated with the potential predisposition to congestive heart failure (where LV systolic or diastolic function is compromised and unable to meet the demands of the body), a poorly recognized concept in sickle cell disease. Furthermore, as with other heart failure patients, consideration of the predisposition to the development fatal ventricular arrhythmias is also possible but not studied.

Prolonged QTc on an electrocardiogram (ECG) is an established risk factor for the development of polymorphic ventricular tachycardia (VT) and sudden cardiac death[11–13]. In limited fashion and mostly in pediatric populations, previous reports in smaller cohorts of sickle cell disease patients have observed the association of prolonged QTc to overall mortality[14–16], an important step toward understanding origins of sudden cardiac death. Moreover, the work from younger populations of sickle cell disease has also reported preliminary associations between prolonged QTc to markers of hemolysis such as aspartate aminotransferase (AST) levels and lactate dehydrogenase (LDH) levels[14]. Additionally, while sickle cell disease origins stem from Mendelian inheritance patterns, patients manifest a wide spectrum of clinical manifestations including the development of hemolytic severity, changes in QTc duration, acute and chronic pain, cerebrovascular disease (CVA), acute chest syndrome (ACS), pulmonary hypertension (PH), and premature or sudden death. Therefore, we sought to further characterize the QTc interval in a large cohort of adults with sickle cell disease and its relationship to outcomes. Furthermore, we sought to prospectively evaluate associations of prolonged QTc with hemolysis by measuring molecular evidence of hemolysis as another marker. Such validation underscores the potential to identify a subset of individuals at risk for poor outcomes and further close the gap on understanding and defining an etiology of sudden death in sickle cell disease.

## Material and Methods

### Patient Population

Subjects with sickle cell disease (demonstrated by high-performance liquid chromatographic separation or gel electrophoresis) from the University of Illinois at Chicago (UIC) and University of Chicago (UC) were examined. All subjects at the UC provided written consent to participate in this study with the approval of the University of Chicago institutional human subjects review board. Patients in the UC cohort were excluded if they were clinically unstable, defined as having vaso-occlusive crisis, acute chest syndrome or unscheduled blood transfusions within 3 weeks of the study. Subjects in the UC cohort were prospectively recruited and had ECGs performed (n = 38). Additionally, in the UC cohort, a subset of subjects underwent phlebotomy (n = 29) with free heme measurements (**Supplemental Methods**), echocardiography (n = 31), cardiac magnetic resonance imaging (n = 32), and where additional plasma was available (n = 17), free heme values were assessed same day as the when the ECG was performed.

With the approval of the University of Illinois at Chicago institutional human subjects review board, UIC subjects were evaluated with retrospective chart review of the ECG (n = 224) and subsets with echocardiographic (n = 126) and laboratory data (n = 146). For each subject, the latest available ECG in normal sinus rhythm were included up until Dec 31, 2014 and categorized as originating from an inpatient or outpatient setting.

For both cohorts, subjects were followed for vital status assessment up to December 31^st^, 2014 using social security index, phone calls and review of electronic medical records. All subjects over the age of 18 were considered for inclusion.

### Electrocardiography

ECGs were recorded with a standard 12-lead configuration at 25 mm/s paper speed and 10 mm/MV amplitude, using a commercially available system (Muse, GE Healthcare). Values of heart rate, QT interval, and other intervals (PR, presence of block, premature atrial and ventricular complexes), were taken from the computer-determined analysis of the ECG, with QTc computed using a Bazett formula. QTc prolongation was defined as above 440ms for males, and above 460ms for females.

### Transthoracic Echocardiography (TTE)

TTE data was obtained using standard 2-dimensional, M-mode and Doppler imaging techniques. TTE was performed the same day as the ECG. The images were obtained using an S5 probe connected to an iE33 ultrasound machine (Philips Healthcare, Andover, MA). Digital cine loops were acquired by a single highly experienced sonographer and subsequently assessed offline. Peak tricuspid regurgitation velocity (TRV, m/s) and left ventricular end diastolic dimension (LVIDD, cm) were measured using the American Society of Echocardiography guidelines[17].

### Cardiac Magnetic Resonance Imaging (CMR)

CMR was only performed on and data was only available from patients in the UC cohort. CMR images were acquired using a 1.5-T scanner (Achieva, Philips, Best, Netherlands) using a 5-element phased array cardiac coil as previously described[7]. The CMR study included assessments of cardiac structure and function. Standard long-axis views were obtained, including four-chamber, two-chamber, and three-chamber images. In addition, one series of short-axis slices (8 mm thickness, 2 mm gap) covering the entire heart was acquired. Images were analyzed using commercial software (Philips ViewForum, Best, Netherlands). Short-axis slices were used to measure maximal left and right atrial volumes, left ventricular (LV) and right ventricular (RV) end-diastolic and end-systolic volumes (EDV and ESV), mass, and ejection fraction (EF) by the biplane method of disks (modified Simpson’s rule) [18]. All volumes and masses were indexed for body surface area.

### Statistics

Data is presented as median and interquartile range (IQR). Comparisons between patients who were alive or died were made with a Kruskal Wallis test. Regression analysis was used to identify factors associated with QTc duration. Factors with a *P*<0.05 on univariate analysis were then included in a multivariate regression analysis. Kaplan-Meier survival analysis was performed stratifying QTc at the 75^th^ and 90^th^ percentiles with time of ECG acquisition as time zero, with survival curves assessed by a log rank test. Hazard ratios were determined by Cox proportional hazards. As vital status was not available for all patients in the UC cohort, the two cohorts were not combined for hazard ratio analyses. Associations between free heme and QTc, echocardiographic, CMR and other laboratory values in the UC cohort were assessed by a Spearman correlation coefficient and linear regression. Statistical analysis was performed with STATA (version 14, StataCorp, College Station, TX). Significance was set at *P*≤ 0.05.

## Results

### UIC Cohort

ECGs from a total of 224 patients were analyzed comprising 134 females. The median age of the cohort was 34.6 years at time of ECG [IQR: 25.7, 47.3] (S1 Table). The median time from ECG to last assessment was 835 days, (range 1–10395 days). The majority of subjects were of hemoglobin SS genotype (77%), with 7% as S/β-thalassemia and 16% as type SC. The median QTc was 441ms with an interquartile range of 428-460ms and 90^th^ percentile QTc at 480ms. QTc prolongation was present in 39% of males, and 27% of females. Over follow-up, a total of 19 subjects (8.5%) died (n = 10 females). Patients that died had a higher peak tricuspid regurgitation velocity [TRV, 1.95 (1.00–2.55) vs 2.74 (1.00–3.00) m/s, *P* = 0.008]. QTc (median [IQR]) was similar among patients that died: 441 [427, 457] vs 443 [435, 468] ms (*P* = 0.14).

Univariate regression analysis (Table 1) showed QTc to be higher for inpatient ECGs (*P*<0.001). Additionally, QTc was positively associated to laboratory values commonly found in a state of hemolysis including AST (*P* = 0.031) and hemoglobin (*P* = 0.014). QTc was also associated with elevated peak TRV (*P* = 0.036), and decreased in SC (*P*<0.001) genotypes compared to the SS genotype. Multivariate regression adjusting for variables that were significant with *P*<0.05 in the univariate analysis (Table 1) revealed significant QTc association with inpatient ECG status *P* = 0.003) with a trend for peak TRV (*P* = 0.066).

**Table 1:** Univariate and multivariate regression analysis for QTc in the UIC Cohort.

	Univariate			Multivariate		
	*P* Value	Coef.	95% CI	*P* Value	Coef.	95% CI
ECG as in-patient	<0.001	16.8	10.4, 23.2	0.003	13.0	4.53, 21.4
LVIDD (cm)	0.395	2.77	-3.65, 9.18			
TRV (m/s)	0.036	5.16	0.35, 9.97	0.066	4.27	-0.289, 8.83
AST (U/L)	0.031	0.17	0.16, 0.33	0.223	0.102	-0.063, 0.268
LDH (U/L)	0.762	0.003	-0.016, 0.022			
Total Bilirubin (mg/dL)	0.375	0.70	-0.85, 2.24			
Hemoglobin (g/dL)	0.014	-3.33	-5.96, -0.69	0.260	0.102	-4.97, 1.35
Creatinine (mg/dL)	0.079	2.61	-0.30, 5.52			
Hg SC vs Hg SS	<0.001	-16.0	-24.8, -7.12	0.271	-10.0	-28.0, 7.9

LVIDD, left ventricular internal dimension in diastole; TRV, tricuspid regurgitant velocity; AST, aspartate transaminase; LDH, lactate dehydrogenase, Coef., Coefficient

**Fig 1:**
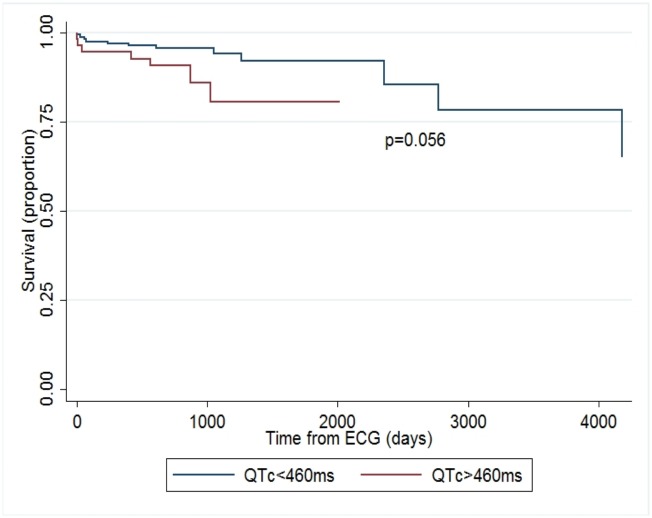
Kaplan-Meier survival curve, QTc of 460ms. Kaplan-Meier survival curves from time of ECG acquisition at the 75^th^ percentile of QTc (460ms) in the UIC cohort.

**Fig 2:**
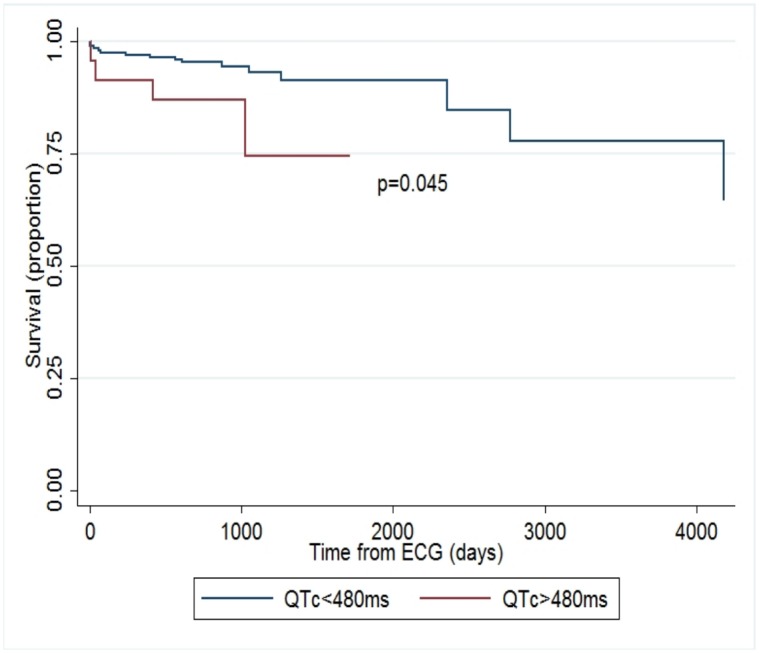
Kaplan-Meier survival curve, QTc of 480ms. Kaplan-Meier survival curves from time of ECG acquisition at the 90^th^ percentile of QTc (480ms) in the UIC cohort.

Univariate Cox regression analysis showed QTc to be related to survival, [hazard ratio (HR) 1.22 per 10 ms interval, *P* = 0.015]. This potential association was also observed at both the 75^th^ percentile of QTc (HR 2.6, *P* = 0.056) and the 90^th^ percentile of QTc (HR 3.19, *P* = 0.045). Adjusting for inpatient ECG status, QTc remained associated with survival [HR 1.26 per 10ms interval, *P* = 0.010]. Multivariate analysis was then performed adjusting for elevated TRV, LDH, and use of hydroxyurea- all known biomarkers for mortality in a continuous model. After adjusting for both LDH and hydroxyurea use, QTc trended toward association with mortality, (HR 1.21 per 10ms interval, CI 0.99–1.48, *P* = 0.062, n = 140). While the QTc hazard ratio was no longer significant after adding elevated TRV and inpatient ECG status to the full multivariate model (HR = 1.23 per 10ms interval, CI 0.98–1.56, *P* = 0.079, n = 122), these additions also further reduced the number of observations. Interestingly, given the proposed association of QTc with hemolysis, a multivariate analysis was performed on the association between QTc and mortality after adjusting for genotype (Hg SS given its increased hemolytic severity compared to Hg SC). The QTc hazard ratio was 1.21 per 10ms interval (CI 1.01–1.44, *P* = 0.037) after adjusting for genotype status (SS versus SC, n = 203). Kaplan-Meier survival curves were also performed and showed a significant decreased survival at both the 75^th^ percentile for QTc (460ms, *P* = 0.048, Fig 1) and 90^th^ percentile for QTc (480ms, *P* = 0.034, Fig 2).

### UC Cohort

The median age in the UC cohort was 38.5 years [32.2, 44.9], and comprised of 23 females. The majority of subjects were of Hg SS genotype (83%), with 8.5% as Hg SC. The median QTc was 439ms with an interquartile range of 422–452 ms and 90^th^ percentile QTc at 466 ms. Characteristics of this cohort including laboratory and CMR findings are described in Table 2. QTc was not significantly associated with TRV, or CMR findings of chamber size. However, QTc was significantly correlated with free heme (r = 0.67, *P* = 0.003).

**Table 2:** QTc, echocardiography, CMR, and laboratory characteristics of the UC Cohort.

	n	Median [IQR]	Correlation Coefficient, (N)*	*P*
QTc (ms)	38	439 [422, 452]	NA	NA
TRV	31	2.36[2.10, 2.60]	0.15, (31)	0.434
MRI-LVEDVi (mL/cm^2^)	32	125[99,139]	0.27, (32)	0.137
MRI-RVEDVi (mL/cm^2^)	32	122[102,138]	0.26, (32)	0.158
Free Heme (mg/mL)	17	196[114, 263]	0.67, (17)	0.003
Hemoglobin (g/dL)	29	8.0[7.2, 9.3]	0.21, (29)	0.274
Creatinine (mg/dL)	29	0.7[0.6, 0.9]	-0.21, (29)	0.285

* Correlation with free heme value, with n referring to the number of subjects compared.

TRV, tricuspid regurgitation velocity; MRI, magnetic resonance imaging; LVEDVi, left ventricular end-diastolic volume indexed to body surface area; RVEDVi, indexed right ventricular end-diastolic volume;

## Discussion

Adults with sickle cell disease present with heterogeneous clinical manifestations, despite Mendelian origins of disease, with a subset of patients who have a greater severity of disease and a higher risk of death. A Kaplan-Meier survival analysis showed increased mortality that was modestly significant for QTc values above the 75^th^ percentile (p = 0.048) and 90^th^ percentile (p = 0.034) in the largest reported cohort of adult sickle cell disease with available ECGs. A Cox regression analysis showed QTc to be related to survival (p = 0.015, unadjusted), which maintained significance when adjusted for inpatient ECG origin (p = 0.010) and for genotype (SS vs SC, p = 0.037). However, this association did not remain significant in a fully adjusted model with the addition of elevated TRV, suggesting the potential for contribution to mortality from this established sickle biomarker. Furthermore, a relationship of QTc to markers of hemolysis was observed in the UIC cohort in univariate analyses to AST and hemoglobin. This relationship to hemolysis was then confirmed in the prospective UC cohort, where QTc was significantly correlated (p = 0.003) to free circulating heme levels.

Our findings support previous investigations of smaller, sickle cell disease cohorts[16]. Prolongation of QTc in sickle cell disease was first reported over 30 years ago, with increased QTc intervals (defined as above 440ms) observed in 9 of 87 hospitalized adult patients[19]. In a cohort of 142 pediatric sickle cell disease patients, QTc was prolonged (defined as greater than 440ms in children and 425ms in adolescents) in 12 subjects[15]. QTc and QTc dispersion were also increased in 60 adult sickle cell disease patients in Nigeria compared to 60 control subjects matched by age and sex, and furthermore, QTc was prolonged beyond 440ms in 61.7% of sickle cell disease patients but not in any of the control subjects[20]. Compared to 25 control subjects[21], the maximum QTc and QTc dispersion were increased in a cohort of 73 (mean age of 18.5 ± 8.0 years) sickle cell disease patients, particularly among those with pulmonary hypertension assessed by echocardiography. In the current investigation, the median QTc of the UIC cohort was similar to these previously reported studies at 441ms, with a 75^th^ percentile measured at 460ms. Based on the potential association with mortality in these studies, in combination, suggest that the thresholds of defining “normal” QTc values in patients with sickle cell disease may be different and potentially, lower than the general population where normal QTc in men is defined up to 450 msec and female up to 470 msec.

Importantly, the current study supports a previously observed relationship between prolonged QTc to mortality. In a prior report of a cohort of 140 adult sickle cell patients who underwent both transthoracic echocardiography and ECG evaluation [16]. the mortality rate was 15% over a 9-year follow-up period and patients at highest risk had persistently prolonged QTc over time and associations with elevated TRV as well as ACS episodes. In that study, a QTc greater than 450ms in men, 470 ms in women was associated with a higher risk of mortality. The current study evaluated a much larger cohort of sickle cell patients which were more enriched with Hb SS genotype patients. We found a QTc > 460ms which is independent of sex that was potentially associated with mortality similar to the Upadhya, *et al* study. Furthermore, the current study expands on this association utilizing both advanced cardiac imaging and biochemical analyses to evaluate association of QTc with structural heart parameters with CMR and hemolysis measuring circulating free heme, respectively.

The current study further supports preliminary suggestions of hemolysis association with prolonged QTc in two independent cohorts. Previous smaller studies have suggested a relationship between prolonged QTc and laboratory values associated with a state of increased hemolysis[14–16]. A relationship of a prolonged QTc to LDH and AST was found in 76 sickle cell children and young adults[14], and QTc was negatively correlated with a lower hematocrit in 62 sickle cell children[22]. In a study of 140 adult sickle cell patients with serial ECGs, a persistently prolonged QTc as well as recurrent acute chest syndromes were associated with mortality[16]. In the current study, while associations with LDH were not observed, there remained a potential relationship between prolonged QTc and mortality when adjusted for inpatient origins of the ECG with weaker associations when adjusted for SS genotype. The significant correlation between measured free heme and QTc prospectively (both obtained on the same day) from the UC cohort further supports a hypothesis that QTc prolongation is related to a state of hemolysis and may be related to the increased risk of mortality. Furthermore, this latter measurement suggests that in addition to the presence of hemolysis, variation in levels of hemolysis may also influence QTc.

A relationship between QTc with estimated right ventricular systolic pressures was also described in the cohort of 76 pediatric and young adults[14]. In contrast, there was no relationship though between QTc and structural heart disease such as left ventricular hypertrophy, evident in 66% of subjects[14]. The current study also did not find an association between QTc and parameters of structural heart disease (left and right atrial and ventricular volumes and biventricular ejection fraction, Table 2) utilizing advanced CMR imaging, considered the gold-standard for cardiac structure and function measurements. The current study also found trends toward a persistent association between elevated TRV and QTc in the UIC cohort (but not in the UC cohort, which may reflect its smaller sample size). Since an elevated TRV has been linked to the presence of increased hemolysis in several sickle cell studies[8, 23] without the presence of pulmonary hypertension, we speculate that the association observed in the current study between elevated TRV and QTc (Table 1) in the UIC cohort may originate from increased hemolysis and not from intrinsic structural heart disease or pulmonary hypertension where the right heart may be remodeled.

Observations of potential increased risk of mortality with prolonged QTc mirror what has been well recognized in the general population. In a study reported nearly 50 years ago[12], patients with recent myocardial infarction and followed with serial ECGs over seven years had a higher risk of sudden death if the QT interval was prolonged. Sudden cardiac arrest was also predicted by QTc in a study of heart failure patients with ischemic cardiomyopathy[24]. From the general heart failure population, myocardial ischemia is as an established risk factor of acquired prolonged QTc. Specifically, the association of acute myocardial ischemia or infarction with QT prolongation is well established[12, 25–27]. Additionally, recently, QTc prolongation has also been reported in patients with non-obstructive coronary disease[28]. Potential mechanisms of QT prolongation in the setting of myocardial ischemia or heart failure may in part be due to an enhanced late sodium current (I_NaL_) which can lead to calcium overload as well as prolongation of the action potential. This can lead to triggered activity with both early and delayed after-depolarizations, and the initiation of ventricular tachyarrhythmias[29]. Based on these studies, previous case reports of sudden cardiac death in sickle cell disease[30], and our previously reported observation of myocardial microvascular disease in sickle cell disease[7], we speculate that the presence of multiple lifetime episodes of hemolytic and vascular occlusion crises, may in part, contribute to increasing coronary microvascular dysfunction, QTc prolongation, and potential association to sudden death in sickle cell disease. Furthermore, the loss of association between QTc and mortality after adjusting for elevated TRV further emphasizes the potential for significant contribution of hemolysis to this relationship. Additional investigation is needed in larger cohorts to determine if sickle cell patients with impaired myocardial reserve have a longer QTc.

While the current study supports previous suggestions of increased mortality risk and hemolysis parameters with prolonged QTc, it is important to note our findings of association of QTc with mortality are of modest significance and do not imply a causal mechanism for death. Additionally, associations of QTc with markers of hemolysis in the UIC cohort were limited as only a subset of the cohort had available blood values and these were not obtained on the same day as the ECG. Given the potential for variation in levels of hemolysis in sickle cell patients day to day which can impact LDH and bilirubin levels, the lack of their association with QTc in the UIC cohort after multivariate analysis may also reflect the influences of this potential variability. Additionally, a potential selection bias may also be present as not all patients completed all studies or had a complete list of potential drugs that may have been administered that could have elevated the QTc at the time of measurement. However, administration of many of these drugs (such as anti-arrhythmics) is rare in sickle cell disease and hydroxyurea, one of the most common drugs administered to sickle cell patients, is not known to result in prolonged QTc. Methadone, used to treat chronic pain, is well known to prolong the QTc; however, a previous investigation found no correlation between methadone dose and QTc in sickle cell patients[16]. Finally, data on causes of death was not available which could further empower links between prolonged QTc and cardiovascular mortality.

## Conclusions

In conclusion, in both a large retrospective and prospective cohorts of sickle cell patients, QTc prolongation was associated with indices of increased hemolysis. These characteristics further support the potential association of QTc prolongation to poor outcomes in sickle cell disease based on the current and previously published studies[16]. Future investigations are urgently needed to address the potential role of serial ECGs which may provide greater insight into the development and progression of prolonged QTc as well as evaluations of causes of death to determine whether there is an association between prolonged QTc and cardiovascular-related mortality and sudden cardiac death.

## Supporting Information

S1 Table Clinical, echocardiographic and laboratory characteristics of the UIC cohort. (DOCX)
