# Supplementary material for: Associations of Prolonged QTc in Sickle Cell Disease
Source: PLoS One. 2016 Oct 13;11(10):e0164526. doi: 10.1371/journal.pone.0164526 (PMC5063274; doi:10.1371/journal.pone.0164526)
Supplement: S1 Table — (DOCX) [file pone.0164526.s001.docx]

**Associations of prolonged QTc in sickle cell disease**

Julia H. Indik^1^, Vineet Nair^1^, Ruslan Rafikov^1^, Iwan S. Nyotowidjojo^1^, Jaskanwal Bisla^1^, Mayank Kansal^2^, Devang S Parikh^2^, Melissa Robinson^3^, Anand Desai^4^, Megha Oberoi^5^, Akash Gupta^1^, Taimur Abbasi^6^, Zain Khalpey^7^, Amit R Patel^8^, Roberto M Lang^8^, Samuel C Dudley^9^, Bum-Rak Choi^9^, Joe GN Garcia^1^, Roberto F. Machado^2^, Ankit A. Desai^1^

**Supplemental Data and Methods**

**Heme Assay**

Plasma samples (10 μl) from adult sickle cell patients in the UC cohort were mixed with 5 μl sample buffer (Tris/SDS/β-mercaptoethanol/glycerol without bromophenol blue) and diluted to 30 ul with PBS. The samples were heated at 70°C for 2 min in a dry bath and were separated using gel electrophoresis in 4-20 % Tris glycine gel. After the electrophoresis, the gel was washed in distilled water and was imaged at 600 nm channel (2 minutes exposition time) on Li-Cor Fc image station. Total fluorescence signal from heme was quantified and analyzed on Image Studio 5.0 software.

**S1 Table. Clinical, echocardiographic and laboratory characteristics of the UIC cohort**

|  | **UIC cohort** |
| --- | --- |
| Age at time of ECG (years) | 34.6 [25.7, 47.3] |
| Sex (female, n=) | 224 |
| Hydroxyurea use (n=) | 70 |
| Hg SS (n=) | 171 |
| Hg SC (n=) | 36 |
| Other genotypes (n=) | 16 |
| ECG (in-patient, n=,%) | 128 (58.0%) |
| QTc (ms)* | 441 [428, 460] |

Data presented as median [interquartile range]. Age determined at time of vital assessment. Hg SS, Hemoglobin SS genotype; Hb SC, Hemoglobin SC genotype
